# Delivery of gene targeting siRNAs to breast cancer cells using a multifunctional peptide complex that promotes both targeted delivery and endosomal release

**DOI:** 10.1371/journal.pone.0180578

**Published:** 2017-06-30

**Authors:** Jeffrey D. Bjorge, Andy Pang, Donald J. Fujita

**Affiliations:** 1 Department of Biochemistry and Molecular Biology, Cumming School of Medicine, University of Calgary, Calgary, Alberta, Canada; 2 The Arnie Charbonneau Cancer Institute, Cumming School of Medicine, University of Calgary, Calgary, Alberta, Canada; Helsingin Yliopisto, FINLAND

## Abstract

RNA interference has been used to dissect the importance of individual gene products in various human disease processes, including cancer. Small-interfering RNA, or siRNA, is one of the tools utilized in this regard, but specially-designed delivery agents are required to allow the siRNA to gain optimal access to the cell interior. Our laboratory has utilized two different siRNA-binding delivery peptides containing a polyarginine core, and modified by myristoylation and targeting motifs (iRGD or Lyp-1). A third peptide was designed to assist with endosomal release. Various ratios of the peptides and siRNA were combined and assayed for the ability to form stable complexes, and optimized ratios were determined. The complexes were found to form particles, with the majority having a diameter of 100–300 nm, as visualized by electron microscopy. These siRNA complexes have enhanced protection from nucleases present in serum, as compared to “naked” unprotected siRNA. The particles were internalized by the cells and could be detected in the cell cytoplasm by confocal fluorescence microscopy. In functional assays, peptide/siRNA complexes were shown to cause the knock down of corresponding targeted proteins. The peptide with the LyP-1 targeting motif was more effective at knockdown in MDA-MB-231 breast cancer cells than the peptide with the iRGD motif. Inclusion of the endosomal release peptide in the complexes greatly enhanced the peptide/siRNA effects. Peptide/siRNA complexes simultaneously targeting Stat3 and c-Myc caused a marked reduction in anchorage-independent growth, a property correlated with tumorigenicity. This study demonstrates the ability of a peptide-based siRNA-delivery system to deliver siRNA into breast cancer cells and cause both protein knockdown and suppression of the malignant phenotype. Such peptide complexes are likely to become highly useful siRNA-delivery vehicles for the characterization, and potentially for the treatment, of human cancer.

## Introduction

By harnessing the power of the RNA interference (RNAi) pathway, short-interfering RNAs (siRNAs) have become a versatile and powerful tool for identifying and characterizing the importance of specific gene(s) in disease processes such as cancer [1–3], and also as a potential therapeutic agent for treatment of various diseases [4–6]. In particular, RNAi lends itself well to current approaches emphasizing personalized medicine combined with targeted therapy, as the length of time it takes to design, test, and produce potentially therapeutic siRNAs is much shorter than the time it takes to develop classical pharmaceuticals, and the costs are considerably less. Importantly, almost any gene or combination of genes can be readily targeted for suppression. Despite its promise, one major obstacle that investigators have had to overcome when developing siRNA-based pharmaceuticals has been the delivery of these highly charged molecules to the targeted cells. Many different methods of delivery are currently under development, each with its own set of advantages [7–11]. It is likely that no one delivery method will prevail for all the disease processes being currently targeted by siRNA, as the location of these disease processes varies within the body, and delivery vehicles may intentionally or unintentionally target certain tissue locations with some selectivity [12, 13], and may also show selectivity towards specific cell types within tissues.

Delivery of siRNA to cells typically requires the siRNA to be bound to or encapsulated by a delivery agent. The makeup of the delivery agent is a critical factor influencing the effectiveness of the siRNA, and it must serve multiple roles. The delivery agent must protect its payload from nuclease-mediated degradation during transit, and facilitate delivery of the siRNA to the targeted cells. The delivery agent must then permit the uptake of the siRNA into the cell, and once inside the cell, must allow movement of the siRNA into the cytoplasm, where the siRNA can finally become incorporated into the RNA-induced silencing complex (RISC) and silence targeted genes.

Because of the complexity of the delivery process, simple approaches using simple unifunctional reagents often don’t yield the desired response by failing to fulfill all of the criteria of an efficient delivery agent. Recently, investigators have begun to design multifunctional or modular delivery agents [14], whereby the different materials/components address one or more facets of efficient delivery and the combination fulfills all or most of the properties of an efficient delivery agent.

Many different materials are currently being examined for their utility as delivery agents to facilitate the delivery of siRNA. Some of these include lipids [15], polymers [16], peptides [17], antibodies [18], and aptamers [19]. Each possess unique properties that make them useful candidates in siRNA delivery vehicles, and research groups have utilized components from one or more of these categories to develop specialized agents that fulfill their requirements for delivery.

Peptides are particularly promising as delivery agents, as there is great deal of preexisting knowledge concerning the biological properties of many naturally-occurring proteins and peptides. This knowledge can be employed to design novel peptides that have properties of an efficient delivery vehicle for siRNA. There are also a number of biologically relevant modifications available (ie. acylation, disulfide bonds, and others), that can be used to potentially modify and enhance their delivery properties. Peptides can also be designed to contain targeting moieties, thus allowing targeting to specific tissues or cells.

Cell penetrating peptides (CPPs) are one class of peptides that have great potential as siRNA delivery vehicles [20, 21]. CPPs were originally discovered in two different proteins, the HIV-1 Tat protein [22] and the drosophila Antennapedia protein [23], and contain short amino acid sequences (named Tat peptide and Penetratin, respectively) that possess properties that allow them to cross the plasma membrane. Subsequently, both naturally occurring and synthetic CPPs with a variety of sequences have been characterized, and most are cationic in charge. Often these cationic CPP sequences adopt an amphipathic helical structure as a result of interspersed hydrophobic amino acids [24]. But synthetic modification of the CPPs amino acid sequence has revealed that even simple polyarginine peptides possess the ability to bind to and penetrate the plasma membrane [25]. Polyarginine is of particular interest because it also has the ability to non-covalently interact with siRNA and can transport bound siRNA into the cell interior [26].

Although CPP peptides have the ability to cross the plasma membrane, most lack binding specificity, and bind to negatively charged cell membranes as a consequence of their intrinsic positive charge. The specificity of CPP peptides towards targeted cells can be improved by a variety of means, including coupling to antibodies or aptamers. Additionally, several laboratories have identified short peptide sequences that were shown to target actively growing tumors in animal models. Two of these targeting peptides, LyP-1 and iRGD, appear to bind to endothelial cells within tumors, as well as tumor cells themselves, and can also facilitate the uptake of bound cargo, including siRNA, directly into tumor cells [27–29]. LyP-1 and iRGD have been found to be effective at selectively targeting tumors of many different types, including prostate, pancreatic, breast, and ovary.

In our laboratory, we have examined several peptides based upon the polyarginine CPP core. We have also added several motifs to this core to assist with particle stability, plasma membrane penetration, and cell targeting and utilized a separate “helper peptide” in the complex to improve endosomal release. Our results demonstrate the ability of this multifunctional peptide complex to facilitate the delivery of siRNA into a human breast cancer cell line and knock down two important genes involved in its transformed growth properties. These results suggest that appropriate strategies utilizing peptide-mediated delivery of siRNA may have considerable promise in the development of future therapeutics for breast cancer and other diseases.

## Materials and methods

### Cells and experimental reagents

The human breast cancer cell line MDA-MB-231 and was obtained from the American Type Culture Collection and were routinely cultured in Dulbecco’s modified Eagles medium containing 10% fetal bovine serum and Gibco antibiotic/antimycotic.

The synthetic peptides were synthesized by GenicBio Shanghai. The sequence of myr-R9-iRGD is myr-d(RRRRRRRRR)GGGGKCRGDKGPDC, myr-R9-LYP is myr-d(RRRRRRRRR)GGGGKCGNKRTRGC, and E9 is GLFEAIEGFIENGWEGMIDGWYGGGGEEEEEEEEE. In the peptide sequences, myr refers to myristate and d refers to D-amino acids. Both myr-R9-iRGD and myr-R9-LYP have internal disulfide bonds. In some experiments, fluorescent versions of the myr-R9-iRGD and myr-R9-LYP were used, and these peptides were synthesized with tetramethylrhodamine attached to the primary amino-group of the lysine immediately adjacent to the 4 glycine linker.

Single stranded RNA for STAT3, cMyc, and control siRNA was synthesized by the University of Calgary Core DNA/RNA facility (Dr. R. Pon) and annealed in RNA buffer (6 mM Hepes pH 7.5, 20 mM KCl, and 0.2 mM MgCl_2_) to form double stranded siRNA (20 μM) with 3′ 2 nucleotide dTdT or UU overhangs. The control siRNA target sequence was AATTCTCCGAACGTGTCACGT. The cMyc siRNA target sequence was AACGATTCCTTCTAACAGA. The STAT3 siRNA target sequence was GGCGTCCAGTTCACTACTA.

### siRNA gel shift assay

16 μl of varying concentrations of siRNA-binding peptides in water were added to 50 μl of PBS and incubated for 5 min. at 20^°^C. In some experiments, the E9 peptide was added for 5 minutes. 5 μl of 20 μM siRNA solution was then added to each tube, mixed briefly on a vortex, and then incubated for 15 min at 20^°^C. 5 μl was then removed from the mixture and added to a tube containing 1 μl of 6X non-denaturing agarose gel loading buffer containing 30% glycerol in water, 0.25% bromophenol blue and 0.25% xylene cyanol FF. The contents were mixed, and then 5 μl loaded onto a 2.5% agarose gel in TBE buffer (0.09 M Tris, 0.09 M boric acid, and 0.002 M EDTA). Following electrophoresis to resolve the mixture, the gel was stained with Sybr Green II RNA stain (In Vitrogen) for 30 minutes and the fluorescent bands visualized using a Fuji LAS4000 Imager.

### Confocal microscopy

MDA-MB-231 human breast cancer cells were plated onto sterile glass coverslips in 6 well dishes and incubated overnight under normal growth conditions. The growth medium was aspirated and the cells incubated with the myr-R9-LyP-1-peptide complexed control siRNA. After 18 hours, the medium was aspirated, the cells rinsed once with serum-free phenol red-free DMEM/PBS (50:50) buffer and stained for 5 min. at 37^°^C in 1 ml of DMEM/PBS containing 1/10,000 dilution CellMask deep red plasma membrane stain (Molecular Probes). The coverslips were then rinsed 2 times with DMEM/PBS and live-mounted onto glass slides in DMEM/PBS using parafilm spacers and sealed with melted paraffin/Vaseline (50:50 w/w). The slides were viewed within 45 minutes using a Zeiss LSM 510 META confocal microscope.

### Transmission electron microscopy

Solutions containing siRNA complexed to peptides were made up as described under siRNA gel shift assays. Small droplets of the siRNA/peptide solution as well as water and a solution of 1% uranyl actetate were spotted onto a piece of parafilm Grids were floated on top of the siRNA/peptide droplet for 2 min and rinsed on a water droplet for 30 sec. The grid was then transferred to the uranyl acetate droplet for 30 sec and then another 30 sec rinse on a water droplet. Excess liquid was then gently blotted from the grid after each step and the grid was viewed using a Hitachi H-7650 transmission electron microscope.

### Serum stability measurements

siRNA was either diluted in 25 μl PBS alone or diluted in PBS containing the siRNA-binding peptide and E9 peptide at the optimal binding ratios. Fetal bovine serum was then added to each of the 2 tubes and the tubes incubated for various lengths of time at 30^°^C. At each time point, a 10 μl sample of the mixture was removed and added to a tube containing 3 μl Pronase (Sigma) (10 mg/ml in PBS with 0.25% SDS and 50 mM CaCl_2_) and further incubated for 5 min at 30^°^C. This step was necessary to degrade the peptides so that they would no longer bind to the siRNA. 4 μl of the Pronase-digested mixture was mixed with 16 μl of urea gel loading buffer (8 M urea, 5 mM Tris, 20 mM EDTA, 0.25% xylene xyanol FF), heated to 95^°^C for 5 min., and then loaded onto a 20% polyacrylamide gel containing 7 M urea and TBE buffer.

### Protein knock-down experiments

MDA-MB-231 human breast cancer cells at 70% confluency in 6 well dishes were either incubated with siRNA complexed with siRNA-binding peptides or with the commercial transfection reagent Lf-2000 (Invitrogen).

When using peptides to form complexes with siRNA, 8 μl of siRNA-binding peptides (500 μM stock) were initially diluted into 100 μl PBS, mixed, and incubated for 5 min. In some tubes, 1.6 μl of E9 peptide (500 μM) was then added and mixed, and incubated for 5 min. 10 μl of 20 μM siRNA was added and mixed, and incubated 15 min. This mixture was then added to the cultured cells in 1.7 ml of Dulbecco’s modified Eagles Medium (DMEM) for 2 hours. 1.8 ml of DMEM containing 20% fetal bovine serum (FBS) was then added to each well and the cells incubated for 48 hours before harvesting for western blot analysis.

With Lf-2000, 20 μl of siRNA was complexed with the transfection reagent in the presence of Optimem I medium following the manufacturer’s directions. The mixture was added to the cultured cells in DMEM containing 10% FBS and the cells incubated for 48 hours before harvesting for western blot analysis.

### Soft agar colony forming assay

MDA-MB-231 breast cancer cells were incubated with Stat3 and c-Myc siRNA using either Lf-2000 or the myr-R9-LyP-1/E9 combination as described above, except that Stat3 and c-Myc siRNA was premixed together prior to adding to the Lf-2000 or the peptides. After 24 hours, the cells were detached using trypsin, counted, and 4.6 X 10^3^ cells were suspended in DMEM containing 10% FBS and 0.35% low-melt agarose (Seaplaque). This was layered over top of a pre-hardened layer of 0.5% low-melt agarose in DMEM containing 10% FBS and was allowed to cool and harden at 23 ^o^C. The plates were then incubated at 37^°^C in 5% CO_2_ for 2 weeks. The plates were supplemented with fresh medium by layering 3 mls of the 0.35% agarose/DMEM solution (see above) into each plate every 4–5 days. After 2 weeks, colonies larger than 50 cells were quantitated under an inverted microscope.

## Results

**Fig 1:**
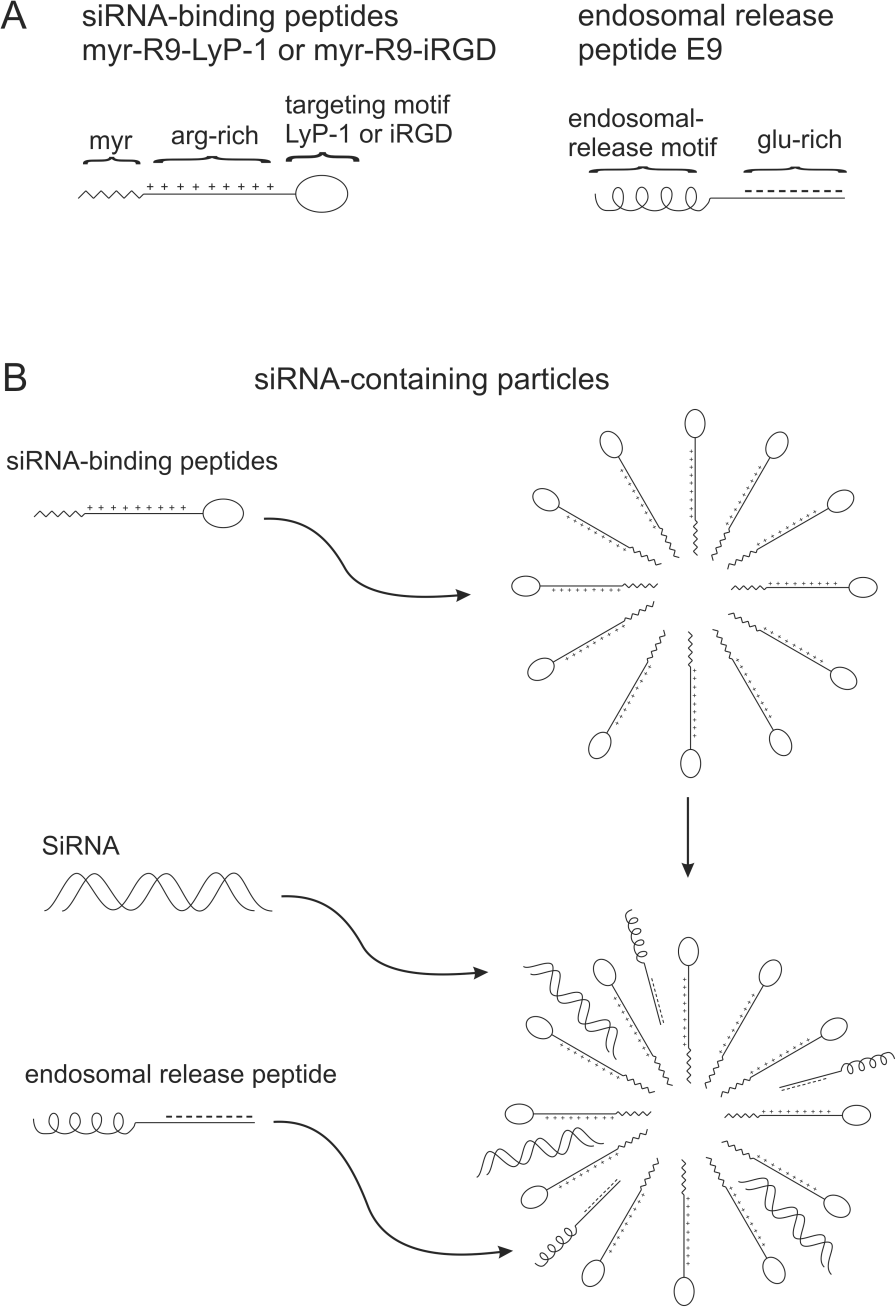
Diagrams of various peptides used in the study and the putative structure of complexes formed between the peptides and siRNA. In Fig 1A, the domain structure of the peptides is shown. The siRNA binding peptides have a myristoylated amino-terminus, followed by an arginine-rich region (R9), and a cell targeting motif (LyP-1 or iRGD). The endosomal release peptide possesses an endosomal release motif flanked by a glutamic acid-rich region. In Fig 1B, the formation of nanoparticles by the siRNA-binding peptide is shown, and the incorporation of siRNA and the endosomal release peptide into the particles after their addition.

We synthesized several peptides that would form multifunctional complexes with properties that would enable the delivery of siRNA into breast cancer cells. These properties included the ability to form small particles in aqueous solutions, to non-covalently bind to and act as a carrier for siRNA, to bind to and be internalized by human breast cancer cells, and finally, to release the siRNA inside the cell to allow suppression of target genes. The domain structure of these peptides can be summarized in Fig 1. Two siRNA-binding peptides, myr-R9-LyP-1 and myr-R9-iRGD were synthesized. They consist of a hydrophobic myristoylated amino-terminus (myr) that facilitates the formation of small particles and assists with the transiting of the particles across cellular membranes. Next, there is a positively-charged arginine-rich segment which allows binding via electrostatic interaction with the negatively charged siRNA and with the plasma membrane. Finally, there is a tumor targeting sequence (either LyP-1 or iRGD) that targets the peptides to cells possessing complementary binding sites. LyP-1 and iRGD were originally identified using phage display library screening [27, 28] and have been shown to target a variety of tumor types [28–30]. A third peptide, an endosomal-release peptide (E9), was synthesized to contain a domain capable of mediating intracellular endosomal release and nine negatively-charged glutamic acid residues, which facilitate the electrostatic interaction with the siRNA-binding peptides.

**Fig 2:**
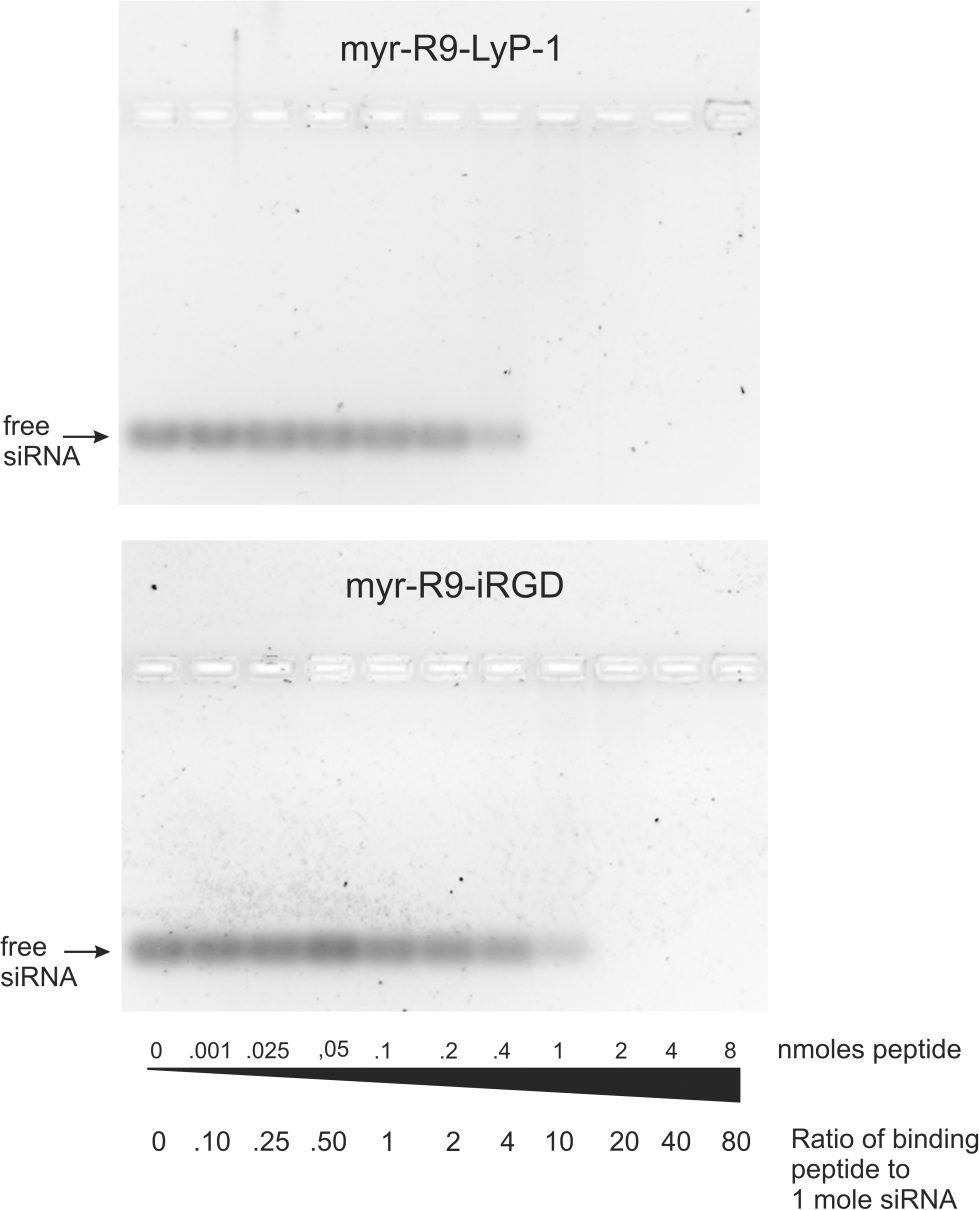
siRNA binding to the siRNA-binding peptides myr-R9-LyP-1 and myr-R9-iRGD as measured by an agarose gel shift assay. 0.10 nmoles of siRNA was incubated with various amounts of two different siRNA-binding peptides, myr-R9-LyP-1 or myr-R9-iRGD. Following the incubation, the free and bound siRNA was separated on a 2.5% non-denaturing agarose gel. The gel was stained with Sybr Green II RNA stain, and the resulting fluorescent bands were visualized with a Fuji LAS4000 Imager. The results presented are representative of three independent experiments.

We initially tested the ability of the siRNA-binding peptides to bind to siRNA using a gel shift assay. Free siRNA can be resolved on an agarose gel and visualized following staining with Sybr Green II dye. When increasing concentrations of the siRNA-binding peptides are coincubated with the siRNA, the siRNA binds to the peptides and its mobility is retarded and it no longer resolves in the gel (Fig 2). Both siRNA-binding peptides tested exhibited this ability, although myr-R9-LyP-1 fully shifted the siRNA at a ratio of 10:1 (peptide:siRNA), whereas myr-R9-iRGD required a higher concentration, and was only able to fully shift the siRNA at a ratio of 20:1.

**Fig 3:**
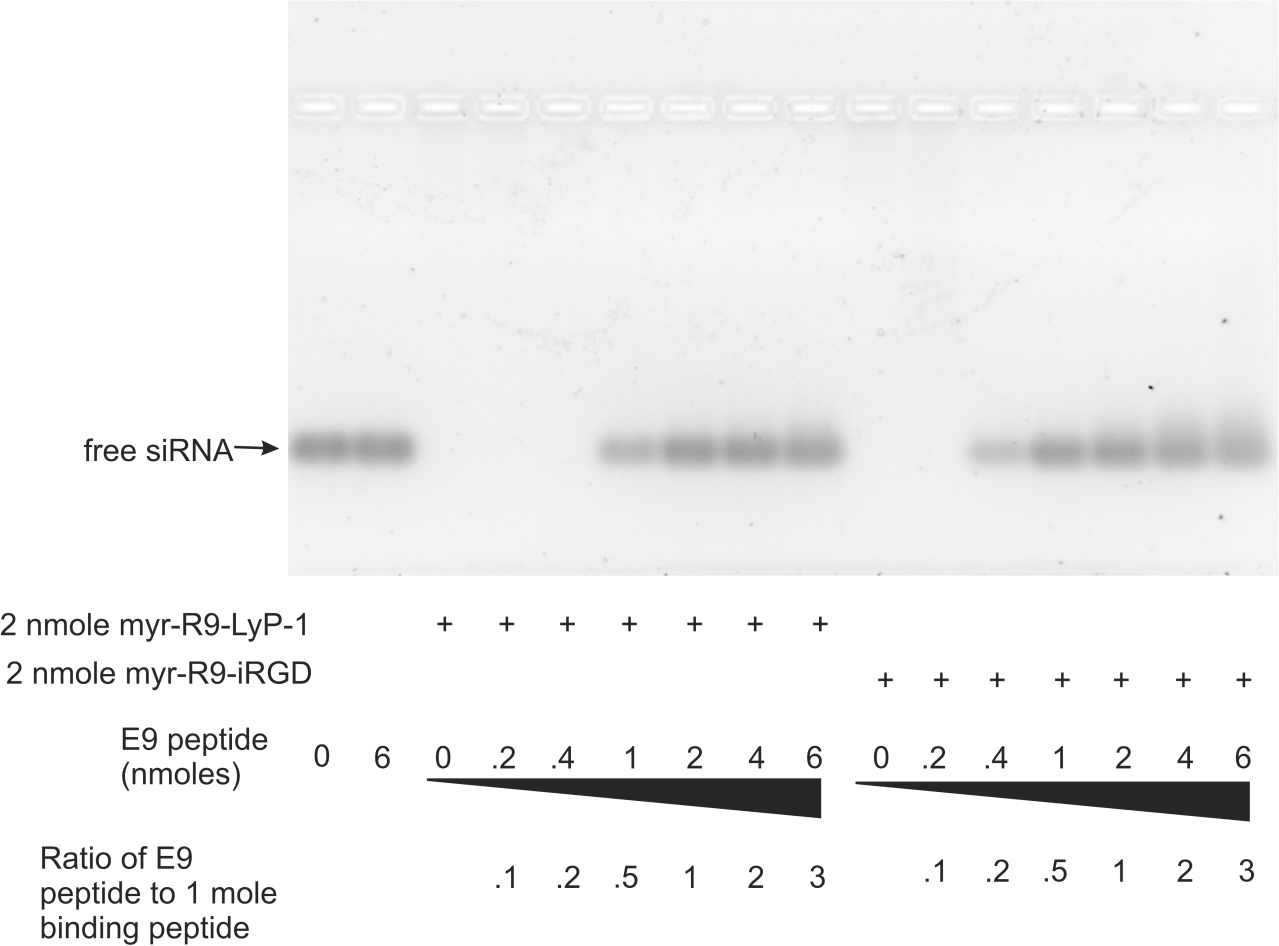
Optimization of the binding of peptide E9 to the siRNA-binding peptides. Various amounts of peptide E9 were added to the siRNA-binding peptides myr-R9-LyP-1 and myr-R9-iRGD, followed by 0.10 nmoles of siRNA. Following 15 minutes of incubation, the free and bound siRNA was separated on a 2.5% non-denaturing agarose gel. The gel was stained with Sybr Green II RNA stain, and the resulting fluorescent bands were visualized with a Fuji LAS4000 Imager. The results presented are representative of three independent experiments.

We also tested whether we could include additional “cargo” in the binding peptide/siRNA complex without causing significant disruption. In particular, we wanted to examine if the inclusion of a peptide capable of facilitating endosomal release might assist with the overall efficiency of gene knockdown. To do this, we utilized a previously characterized peptide sequence [31], INF-7 (derived from the HA-2 subunit of influenza virus hemagglutinin), that possesses the property of enhancing endosomal release. To improve the ability of INF-7 to bind the positively-charged siRNA-binding peptide, we added 9 negatively charged glutamic acid residues to its carboxy-terminus (E9 peptide). We then examined what ratios of this peptide could be included in the complex before it would start to compete with and displace the siRNA; a situation which we wished to minimize. In this experiment, the ratios of siRNA and peptide myr-R9-LyP-1 or myr-R9-iRGD were chosen such that all of the siRNA would be bound in the complex. Fig 3 shows that increasing the amount of the E9 peptide did lead to a gradual displacement of the siRNA from the peptide particle complex, with complete displacement seen with 2 and 1 nmoles E9 peptide, using peptides myr-R9-LyP-1 and myr-R9-iRGD, respectively. The highest amount of E9 peptide that did not cause any detectable displacement was 0.4 and 0.2 nmoles, for peptides myr-R9-LyP-1 and myr-R9-iRGD, respectively. The E9 peptide alone did not appear to interact with the siRNA (lane 2).

**Fig 4:**
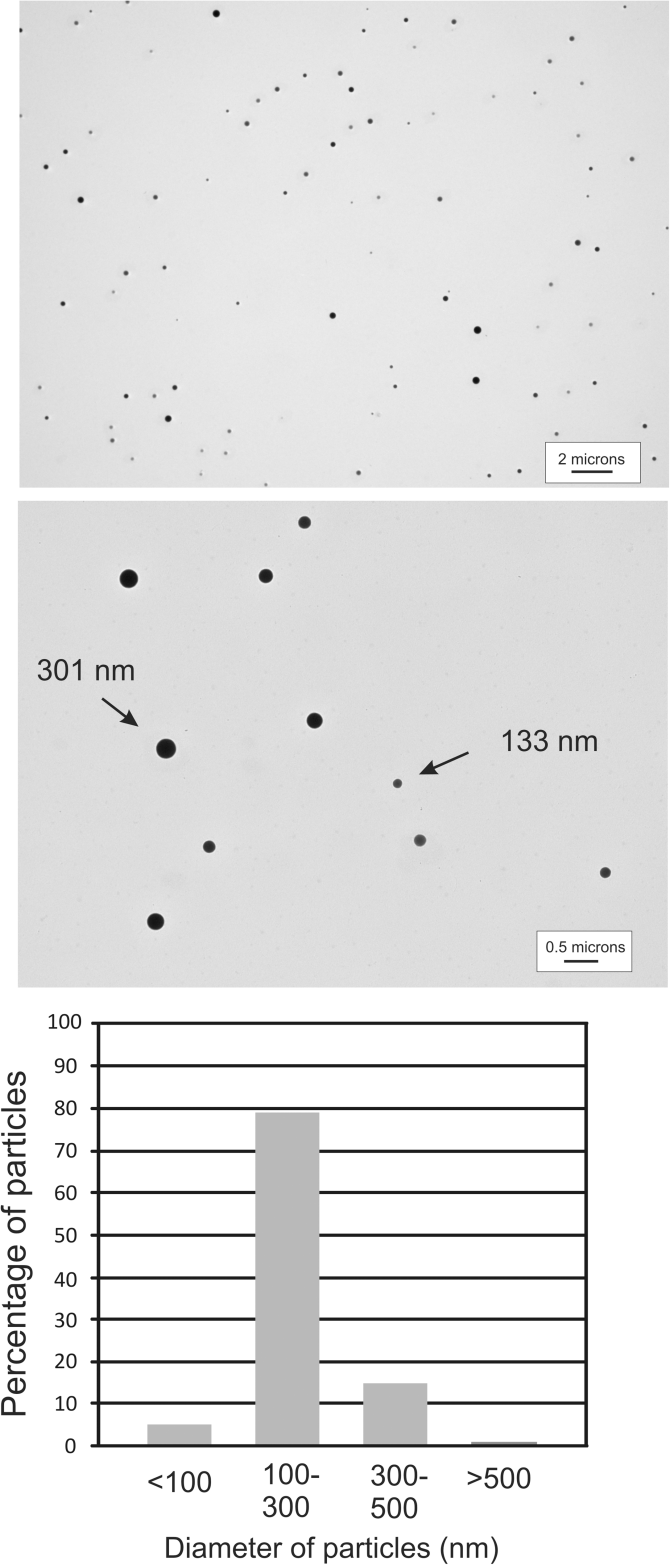
Visualization of the peptide/siRNA peptide complexes by electron microscopy. Peptide/siRNA complexes were stained with uranyl acetate and visualized on a transmission electron microscope. Numerous stained spherical particles were observed. Two representative fields are shown, one at 1500X (top) and 5000X (bottom) magnification. The particles from two separate fields at 1500X and 1200X magnification were measured (see S1 Fig) and the particle distribution is shown in the graph.

Our peptide/siRNA complexes were presumed to form small particles based upon their sequence (providing a hydrophobic core and hydrophilic exterior) and reports from other investigators using similar approaches to design siRNA-binding peptides. To confirm this, we examined an aqueous solution of our siRNA-binding peptide complexed to siRNA by transmission electron microscopy. Following application of the particles to a copper grid and staining with uranyl acetate, we observed numerous small electron-dense particles (Fig 4, upper and middle panels and S1 Fig). These particles were quite similar in size, with most ranging from approximately 100 nm to 300 nm in diameter. The range of particle size is important, because particles smaller than 300 nm have the ability to escape leaky newly-formed tumor-associated blood vessels to gain access to the tumor mass [32]. Measurement of the particles from two separate photographic fields (S1 Fig) indicated that 79% of the particles ranged from 100–300 nm in diameter, with 5% falling below 100 nm, 15% ranging from 300–500 nm, and 1% were larger than 500 nm (Fig 4, lower panel). A diagram illustrating the putative structure of these particles is shown in Fig 1.

**Fig 5:**
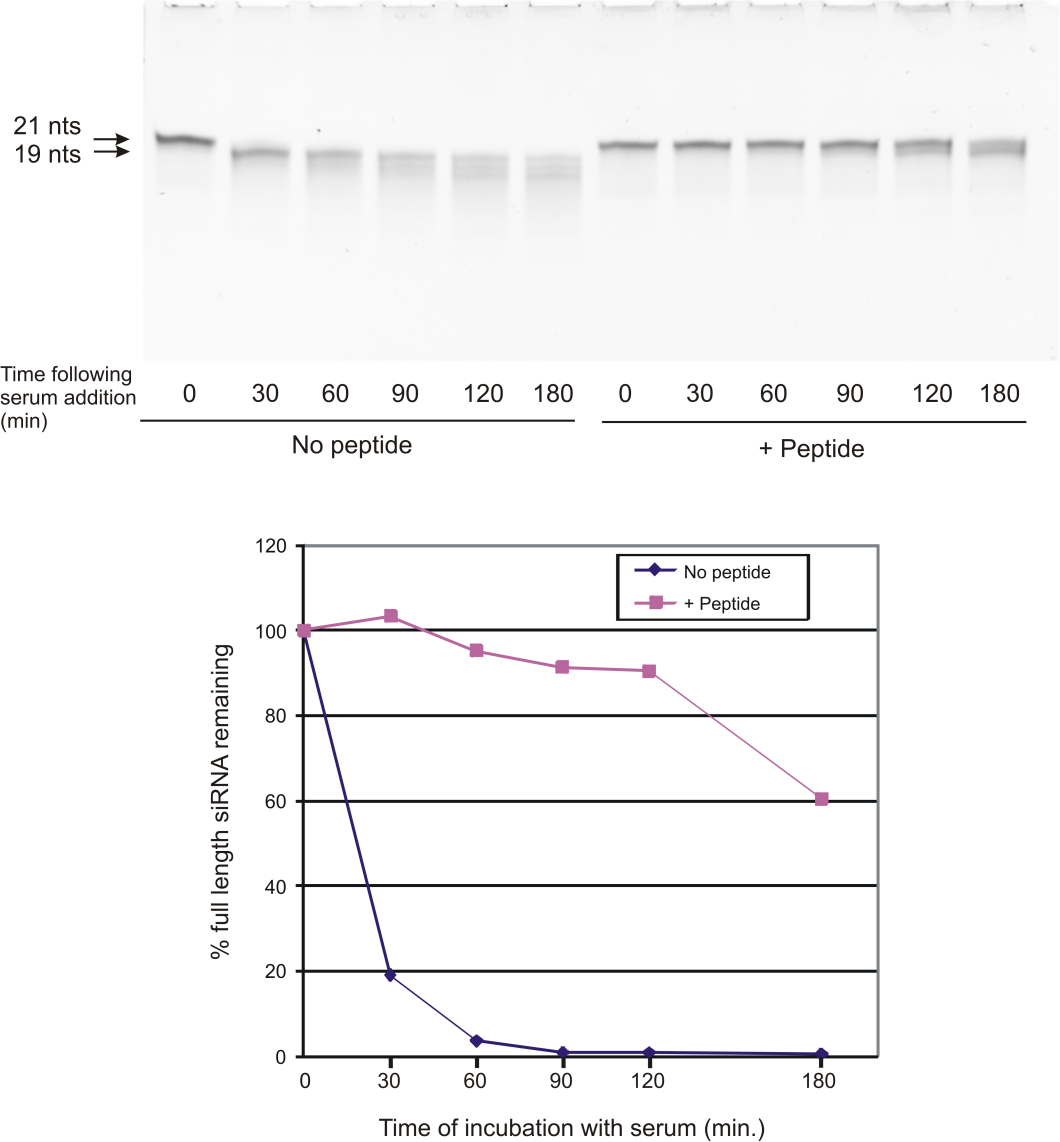
Binding to the targeting peptide protects the siRNA from serum-mediated degradation. Control non-targeting siRNA was either untreated (No peptide) or incubated with myr-R9-LyP-1 peptide (+ peptide) and E9 to form peptide/siRNA complexes. The unbound or bound siRNA were then incubated in the presence of bovine serum for the indicated lengths of time and then separated on a 7M urea gel. The siRNA bands were visualized by staining with Sybr Green II (upper panel). The full-length 21 nucleotide band was quantitated using ImageQuant software (GE) and the results shown in the lower panel. The results presented are representative of three independent experiments.

siRNA must be protected from degradation by nuclease activity prior to becoming incorporated into the RISC complex inside the cell. We tested whether siRNA was protected when complexed with peptide as compared to naked siRNA after exposure to serum, using an assay in which the siRNA is resolved on an 8M urea polyacrylamide. Under these conditions, the 21 nucleotide double-stranded siRNA is separated into single strands that are resolved based upon strand length. (Fig 5). Naked intact Stat3 siRNA incubated with bovine serum became almost completely degraded to a slightly smaller fragment within 30 minutes of incubation, as evidenced by a small downward shift in the siRNA band as compared to undegraded siRNA (0 time). This smaller siRNA band co-migrated with a 19 nucleotide marker, suggesting that the single-stranded 3’ 2 nucleotide overhangs of the double-stranded siRNA were being lost. At 60 and 90 minutes, there was a further reduction in the staining intensity of this lower molecular weight band, and the appearance of faint bands of smaller sizes. When the Stat3 siRNA was pre-complexed with peptide, little degradation of the intact siRNA was seen for at least 90 minutes. By 120 and 180 minutes, some degradation of intact siRNA had occurred, as evidenced by the appearance of a band slightly below the intact band.

**Fig 6:**
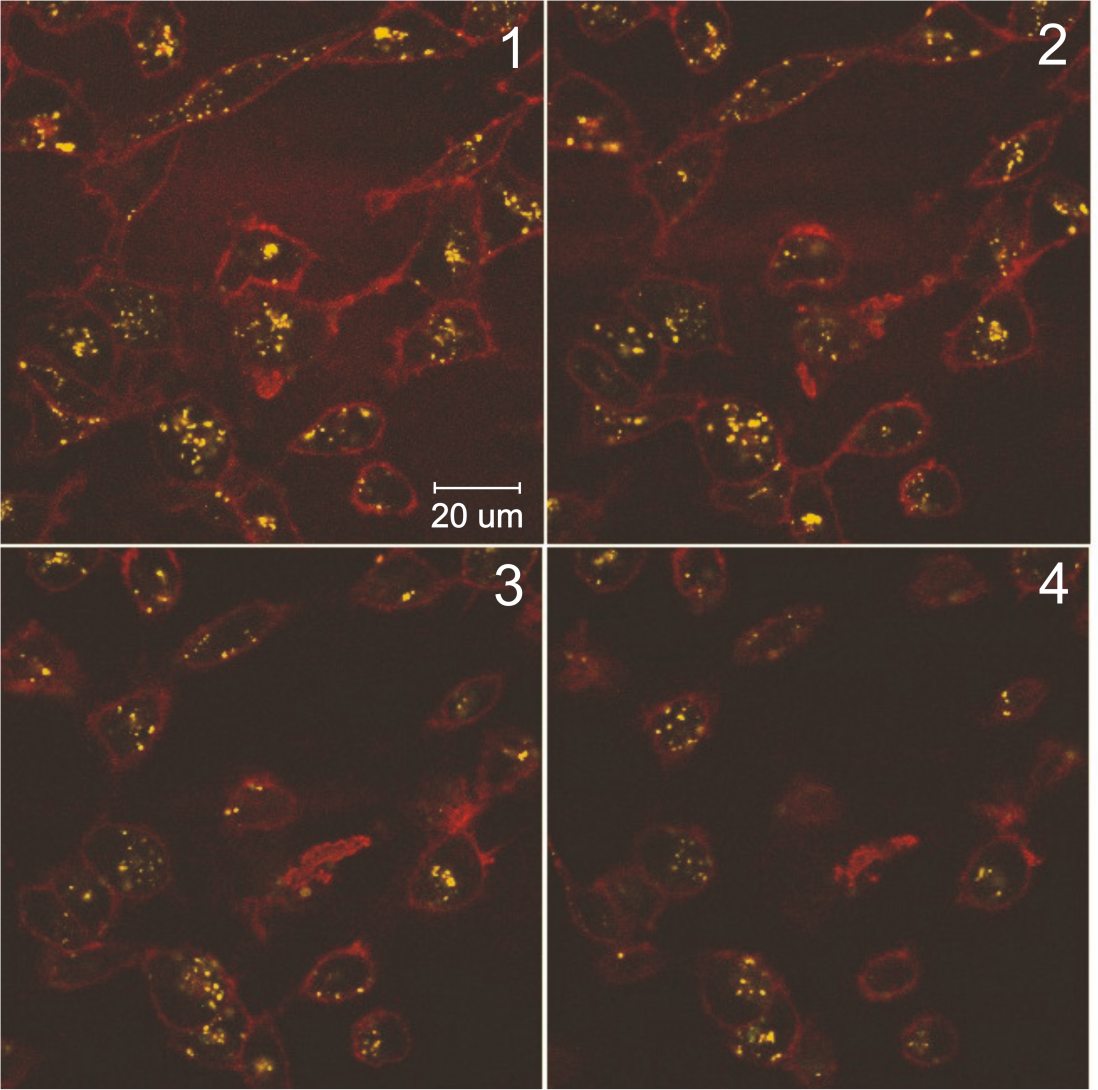
Confocal microscopy of MDA-MB-231 cells incubated with myr-R9-LyP-1/siRNA complexes. Peptide/siRNA complexes were formed as described in the material and methods using fluorescently-labelled siRNA-binding peptide. The complexes were incubated on MDA-MB-231 cells for 18 hours, and the cells were then live-mounted onto glass slides. The slides were viewed by confocal microscopy, and serial sections through one field of cells are shown (panels 1–4). The fluorescently-labeled siRNA-binding peptide is shown in yellow, and the plasma membrane is shown in red (Cell Mask plasma membrane stain). The results presented are representative of three independent experiments.

In experiments with live cells, we initially examined whether the peptide/siRNA complexes would be internalized into cells. We utilized a fluorescently-labeled version of the two targeting peptides to examine the location of the peptide/siRNA particles following incubation of MDA-MB-231 cells with the peptide/siRNA complex. After 18 hours incubation of the MDA-MB-231 cells with the fluorescent peptide/siRNA complex, numerous small fluorescent particles were observed by confocal microscopy to be present within the cytoplasm of MDA-MB-231 cells (Fig 6). The distribution and sizes of the particles varied somewhat from cell to cell, but the vast majority of the cells contained fluorescent particles. They had a fairly even distribution throughout the cytoplasm, but were not always seen in every confocal plane taken through the cell. A few cells showed localization of the fluorescent particles more towards the inner surface of the cell membrane but these were in the minority. Also, a few larger fluorescent particles or aggregates of particles were observed in some cells.

**Fig 7:**
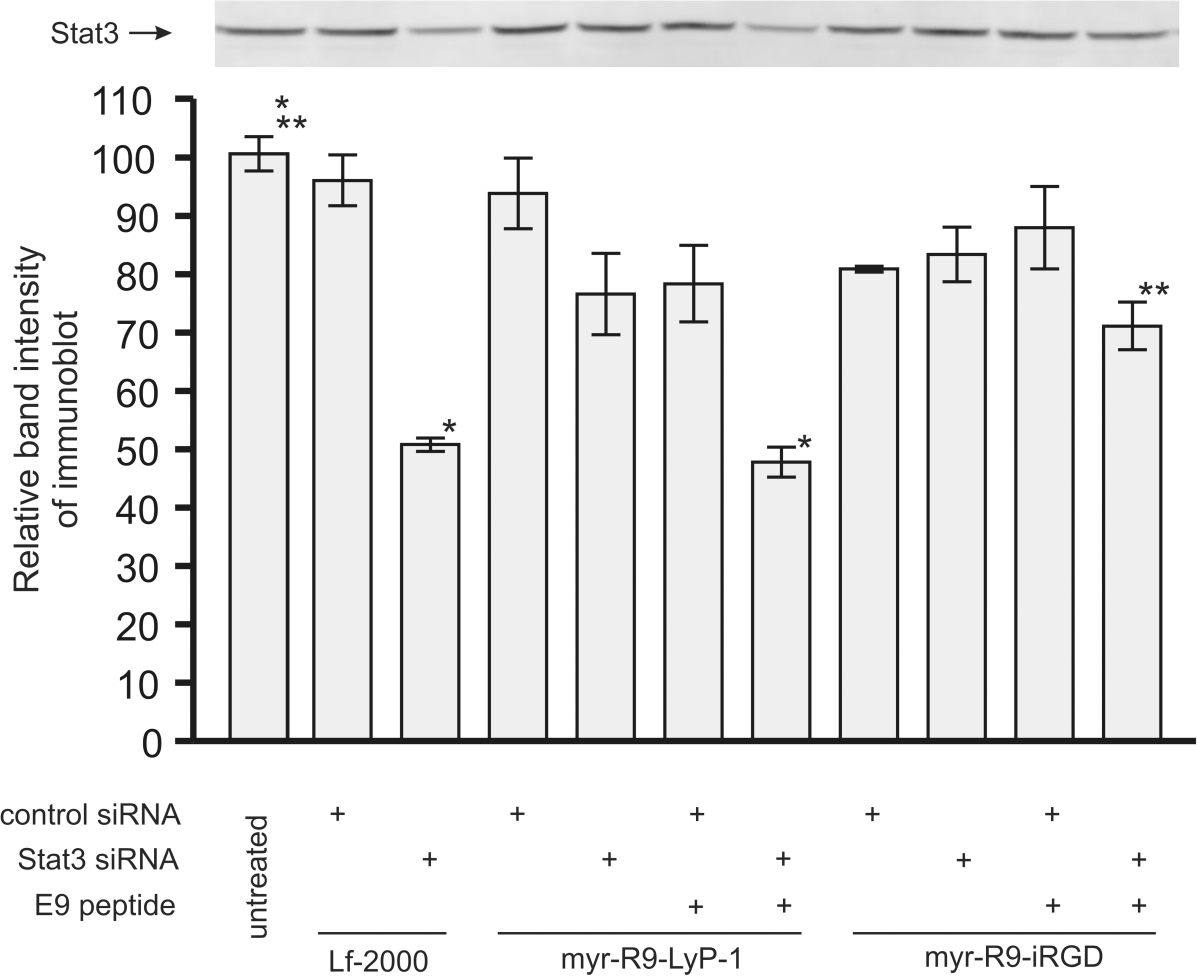
Stat3 knockdown by siRNA delivered to the MDA-MB-231 cells using myr-R9-LyP-1 or myr-R9-iRGD. Peptide/siRNA complexes were formed with optimized ratios of siRNA binding peptide, siRNA, and endosomal release peptide, and incubated on the cells overnight. After 48 hours, cell extracts from the MDA-MB-231 cells were examined for Stat3 protein expression by Western blotting. A representative visualized Stat3 blot is shown along with a bar graph displaying the quantitated results derived from three independent experiments (means +/- SEM; results normalized to 100% relative to the mean of one triplicate set of untreated samples). Statistical analysis consisted of Analysis of Variance and Tukey's pairwise comparison. Significant differences are indicated (* p < 0.001 and ** p < .01).

After determining a set of set of conditions that facilitated the formation of stable complexes of siRNA, binding peptide, and endosomal release peptide, we tested the ability of these complexes to knock down Stat3 in the human breast cancer lines MDA-MB-231 (Fig 7 and S2 Fig). Cells were incubated with the complexes for 48 hours and then the protein expression of Stat3 was examined by western blotting. As expected from previous experiments, Stat3 siRNA was capable of knocking down Stat3 protein expression in the MDA-MB-231 using the commercial transfection reagent Lipofectamine-2000 (lane 3). In the absence of E9 peptide, there was no statistically significant knockdown of Stat3 observed with either myr-R9-LyP-1 or myr-R9-iRGD. Inclusion of E9 in the transfection complex greatly enhanced the Stat3 knockdown, with the magnitude of knockdown observed with myr-R9-LyP-1 similar to that seen with Lipofectamine-2000 (lanes 7 vs 3). Myr-R9-LyP-1 seemed to be the more effective of the 2 peptides, causing a 52% protein knock down of expressed Stat3 protein levels, whereas myr-R9-iRGD elicited a 29% protein knock down.

**Fig 8:**
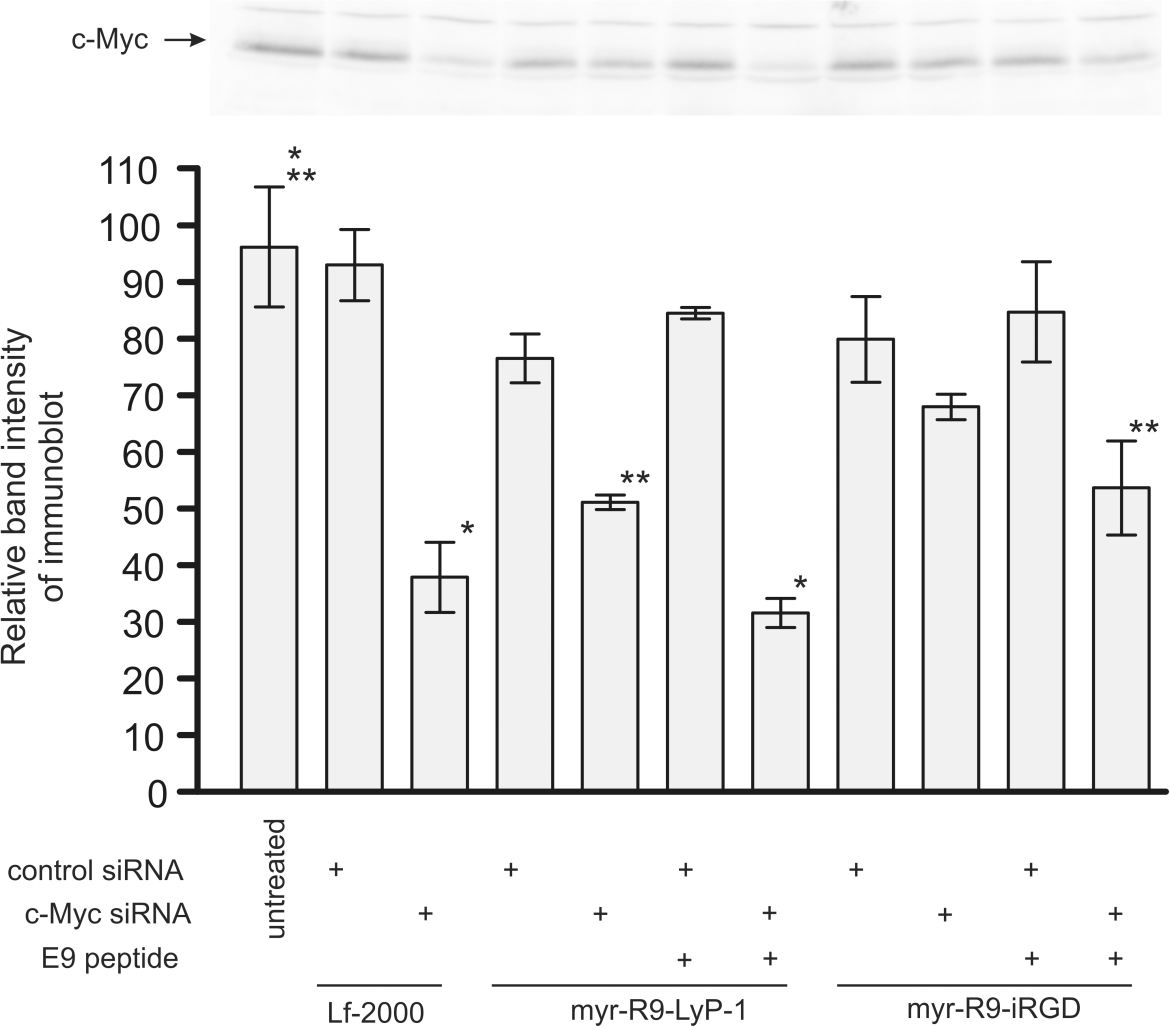
c-Myc knockdown by siRNA delivered to the MDA-MB-231 cells using myr-R9-LyP-1 or myr-R9-iRGD. Peptide/siRNA complexes were formed with optimized ratios of siRNA binding peptide, siRNA, and endosomal release peptide, and incubated on the cells overnight. After 48 hours, cell extracts from the MDA-MB-231 cells were examined for c-Myc protein expression by Western blotting. A representative visualized c-Myc blot is shown along with a bar graph displaying the quantitated results derived from three independent experiments. (means +/- SEM; results normalized to 100% relative to the mean of one triplicate set of untreated samples). Statistical analysis consisted of Analysis of Variance and Tukey's pairwise comparison. Significant differences are indicated (* p < 0.001 and ** p < .01).

A second siRNA, c-Myc siRNA, was also tested in the MDA-MB-231 cells (Fig 8 and S3 Fig). Similar to the previous results with Stat3, myr-R9-LyP-1 accompanied by the E9 peptide gave the largest knockdown (68% knockdown). In the absence of E9 with myr-R9-LyP1, and consistent with our previous results with Stat3 siRNA, there was a reduced effect (49% knockdown). We observed a 46% knockdown of c-Myc protein using myr-R9-iRGD and E9, but it was not as effective as myr-R9-LyP1.

**Fig 9:**
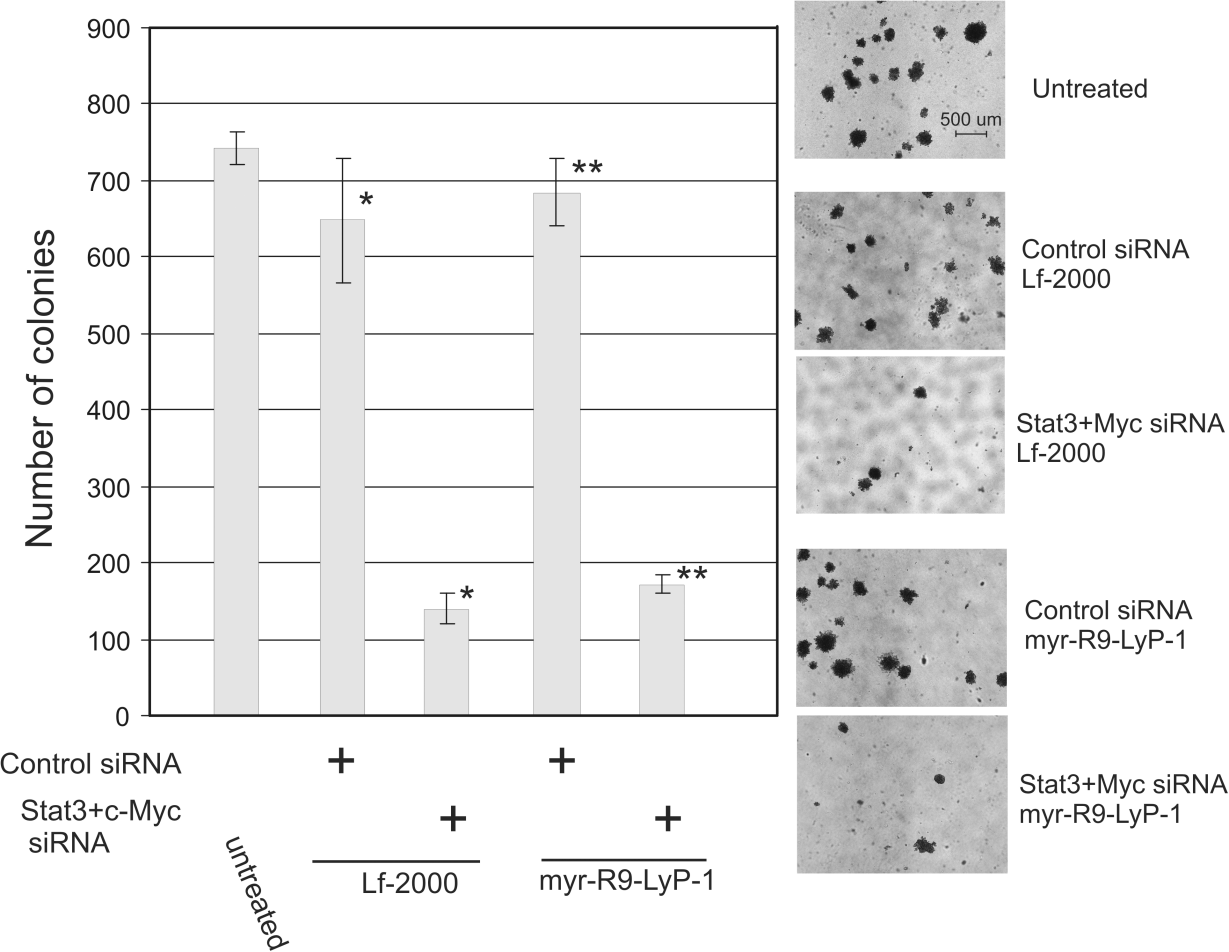
Inhibition of soft agar colony formation by the combination of Stat3 and c-Myc siRNA complexed with myr-R9-LyP-1 and E9 peptides. MDA-MB-231 cells were either left untreated or incubated with control siRNA or a combination of Stat3 and c-Myc siRNAs complexed with either Lf-2000 or myr-R9-LyP-1 and E9 peptides. After 24 hours, the cells were detached with trypsin and replated in soft agar as described in the materials and methods. After 2 weeks, cell colonies larger than 50 cells were quantitated and the results shown. The height of each bar is calculated from the mean of triplicate measurements +/- 1 S.D, and the data analyzed by Analysis of Variance and Tukey's pairwise comparison. Significant differences are indicated (* and ** p < .001). A representative photomicrograph is shown from each condition.

To test whether the peptides could elicit a biological response as a result of the protein knockdown, we utilized a soft-agar colony forming assay which measures the ability of cells to form anchorage-independent colonies in low-melt agarose. The ability to suppress anchorage-independent growth in soft agar is a characteristic of transformed or cancer cells that is highly correlated with tumorigenicity [33]. Stat3 combined with c-Myc siRNA has previously been demonstrated in our lab to cause a large reduction in soft-agar colony formation in MDA-MB-231 cells (unpublished observation). We therefore compared the ability of the optimized combination of peptides (myr-LyP-1 and E9) to that of Lf-2000 in this biological assay. The results show that treatment of MDA-MB-231 cells with the combination of Stat3 and c-Myc siRNA and complexed with myr-R9-LyP-1 greatly reduces the colony forming ability of the cells (74% reduction compared to control siRNA treatment), and the magnitude of the reduction was similar to that seen with Lf-2000 (Fig 9 and S4 Fig). The control non-targeting siRNA did not affect the colony forming potential of MDA-MB-231 cells versus untreated cells with either Lf-2000 or myr-R9-LyP-1.

## Discussion

In this study, we have examined the ability of synthetic peptides to complex with and deliver a siRNA cargo to human breast cancer cells. We have designed these peptides utilizing currently available information and have utilized and combined peptide sequences/motifs and modifications from several sources that we felt would best facilitate the many steps in the delivery process. These steps include the binding of siRNA, the safe transport of the peptide/siRNA complex to cells, the uptake of the peptide/siRNA complex into the cytoplasm of the cells, and finally the release of the siRNA from the complex and incorporation into the RISC silencing complex.

The two binding peptides we have examined, myr-R9-LyP-1 and myr-R9-iRGD, were both designed around the core sequence consisting of 9 arginines possessing a strong positive charge. Polyarginine, and modified forms of polyarginine, have been identified as efficient delivery vehicles for cargo such as siRNA [26, 34–37]. These charged amino acids serve 3 purposes which benefit the delivery of the siRNA to cells. The first purpose is to provide a binding region for the negatively charged siRNA. The second is to provide a charged hydrophilic surface to facilitate the formation of nanoparticles. The third purpose is to enhance the ability of the particles to associate with and be internalized into cells.

The endosomal release peptide, E9, consists of a previously characterized peptide sequence, INF-7 [31], to which we added a short anionic tail. The INF-7 peptide sequence has the previously documented ability to disrupt membranes in a ph-dependent manner, thereby allowing molecules complexed with it, such as nucleic acids, to leak out of endosomes during their acidification [31]. The anionic tail was added to enhance the ionic interaction of E9 with the cationic binding peptides.

During the optimization of the complex formation between the binding peptides myr-R9-LyP-1 and myr-R9-iRGD and siRNA, the goal was to find conditions where all the siRNA added to the peptides became fully complexed as measured by the absence of free siRNA. It would also be advantageous if the positively charged nature of the particles was preserved, as this would likely assist in the binding and internalization of the particles and their siRNA cargo. To fulfill these requirements, the association between peptides and siRNA was examined after complexing different ratios of the constituents by separating the bound and free siRNA on an agarose gel. siRNA that associated with the peptide particles would no longer migrate as free siRNA and would either not enter the gel due to the size of the particle complexes or be severely retarded. Based upon the results of these experiments, were able to choose optimal ratios of peptide to siRNA. Additionally, before testing if the E9 peptide would assist with endosomal release, we tested different ratios of this peptide to ensure that it wouldn’t disrupt the binding peptide/siRNA complex, and were able to choose an optimized ratio of E9.

Examination of the siRNA/peptide complexes by transmission electron microscopy revealed that the complexes formed particles in aqueous solution. We were also able to obtain an estimate of the size distribution of the particles by measuring the diameters of a subset of the particles visualized using this technique. We found that most of the particles ranged from 100–300 um in diameter. This particle size is in a range that enables particles to escape from newly formed blood vessels adjacent to and within a growing tumor mass and accumulate in these regions [32].

One of the advantages of packaging the siRNA into particles is that it would likely have a protective function against nuclease-directed degradation. We tested this hypothesis by incubating both naked and complexed siRNA in the presence of bovine serum for different length of time and examining the state of the siRNA following this incubation. Our results indicated a highly significant stabilization of the siRNA when complexed with peptide, with full stability noted out to 90 minutes, and considerable stability past 180 minutes, in the presence of serum. In the absence of peptide, significant degradation of the naked siRNA was noted at our shortest time point of 30 minutes. There seemed to be 2 phases to the degradation of naked siRNA; a rapid phase of degradation that resulted in almost complete conversion to a 19 nucleotide form in less than 30 minutes and a more prolonged phase of steady degradation to shorter fragments. It is likely, from the known structure of the duplex (21 nucleotides in each single strand, with 19 nucleotides base paired between complementary stands and 2 nucleotide single-stranded 3’ overhangs), that this initial loss likely resulted from exonuclease digestion of the 3’ single-stranded ends. Then, with incubation times greater than 30 minutes, a slower progressive loss of the 19 nucleotide double-stranded form likely occurred, with the accompanying appearance of several lower molecular weight forms. Notably, in the presence of peptide, siRNA was largely protected from these initial stages of digestion for at least 120 minutes.

Depending upon the mode of interaction with the cell surface and the mechanism of internalization into the cell, internalized siRNA often ends up in endosomes, where it is destined for degradation [38, 39]. Various strategies have been employed by others to promote the release of the siRNA from this cellular compartment into the cytoplasm, where the machinery for RNAi is located. These include the use of protonable cationic agents that act as “proton sponges” or fusogenic peptides which disrupt the endosomal membrane [38]. In our experiments using binding peptides alone in the absence of the endosomal release peptide, we found very little knockdown with Stat3 siRNA, and a low level of knockdown with c-Myc siRNA. We attribute this difference in knockdown between the two siRNAs in the absence of the endosomal release peptide to be at least partly due to differences in their overall potency at causing protein knockdown, with the Myc siRNA generally causing the highest level of knockdown. However, the addition of the endosomal release peptide E9 to the complex greatly enhanced the extent of knockdown with either siRNA. This suggests the possibility that in the absence of E9, a proportion of the siRNA is being degraded in the endosome, reducing its effectiveness in our biological assays of protein knockdown. The addition of the peptide E9 containing a peptide sequence known to facilitate endosomal escape [31] may allow a larger proportion of the siRNA to escape degradation, thereby facilitating protein knockdown. Further studies will be required to reveal the details of this enhanced response.

Our approach is unique in that we have incorporated the endosomal release sequence into a separate peptide that also binds to the polyarginine motif within the siRNA-binding peptide via a glutamic acid-rich tail. Although it does complete for binding to siRNA-binding peptide as shown by our competition experiment, we have chosen conditions whereby it does not displace significant amounts of siRNA, while still binding at a high enough ratio to provide the biological property of endosomal release.

It was of interest that in the MDA-MB-231 breast cancer cells examined in this study, the myr-R9-LyP-1 peptide gave greater knockdown of Stat3 and c-Myc than myr-R9-iRGD. We did notice a significant difference in the fluorescent signal in cells incubated with the fluorescent derivatives of the binding peptide, with higher fluorescence seen with myr-R9-LyP-1 (results not shown). This may at least partially explain the differences, as more siRNA would likely be also delivered to the cells using myr-R9-LyP-1. It is important to note that the MDA-MB-231 cells express the receptors for the targeting components of our peptides (αv-integrins for iRGD, p32/gC1qR for LyP-1, and Neuropilin 1 for both iRGD and LyP-1, [30, 40, 41], but we do not know their relative abundance on the cell surface and binding affinities for the peptides. But it interesting to note that there are these differences in binding and protein knockdown, and one peptide may have distinct advantages, depending upon the context in which it is used.

After optimizing the conditions for the formation of the nanoparticles containing siRNA, we tested these nanoparticles in a biological assay measuring the ability to suppress anchorage-independent growth and the formation of cell colonies in soft agar. This assay provides a good readout for suppression of the tumorigenic properties of cancer cells [33] and we have previously used it in our laboratory to examine the ability of siRNAs targeting various gene products to inhibit the growth of several breast and colon cancer cell lines [42, 43]. We examined the ability of the most efficient peptide combination in our studies to suppress the ability of the MDA-MB-231 breast cancer cells to form colonies in soft agar using a combination of siRNAs that we have previously shown to inhibit anchorage-independent growth (unpublished observations). We found that the optimized ratios of myr-R9-LyP-1 and E9 gave a high level of inhibition of soft agar colony formation using the siRNA combination targeting Stat3 and c-Myc. Delivery of non-targeting siRNA with the peptides did not significantly affect colony-forming ability over that seen in untreated cells, indicating the lack of toxicity in this context. These results demonstrate the effectiveness of our peptide/siRNA complexes in a biological assay and suggest that such complexes might be useful in future therapeutic applications. It is interesting to note that the MDA-MB-231 breast cancer cells are triple-negative, lacking the receptors for estrogen, progesterone, and human epidermal growth factor receptor 2. Triple-negative breast cancers are particularly difficult to treat with standard radiation and chemotherapeutic agents, and alternate treatment regimens involving targeted inactivation of specific gene products or pathways may become valuable adjuncts to current protocols.

## Supporting information

S1 Fig Visualization of the peptide/siRNA peptide complexes by electron microscopy. The peptide/siRNA complexes were stained with uranyl acetate and were visualized on a transmission electron microscope. Two fields at A) 1500X magnification and B) 1200X magnification were photographed and these pictures are shown. The particles from the 2 images were measured and the numbers of particles that fell within specified size ranges reported (C). The particle size distribution shown in Fig 4 is derived from the measurement of the particles from these two photographs.
(TIF)

S2 Fig Stat3 knockdown by siRNA delivered to the MDA-MB-231 cells using myr-R9-LyP-1 or myr-R9-iRGD. Peptide/siRNA complexes were incubated on the cells overnight. After 48 hours, cell extracts from the MDA-MB-231 cells were examined for Stat3 protein expression by Western blotting. The resulting blots from triplicate experiments are shown (upper panel) and the results from quantitation of the blots (lower panel). The results shown in Fig 7 are derived from these 3 experiments.
(TIF)

S3 Fig c-Myc knockdown by siRNA delivered to the MDA-MB-231 cells using myr-R9-LyP-1 or myr-R9-iRGD. Peptide/siRNA complexes incubated on the cells overnight. After 48 hours, cell extracts from the MDA-MB-231 cells were examined for c-Myc protein expression by Western blotting. The resulting blots from triplicate experiments are shown (upper panel) and the results from quantitation of the blots (lower panel). The results shown in Fig 8 are derived from these 3 experiments.
(TIF)

S4 Fig Inhibition of soft agar colony formation by the combination of Stat3 and c-Myc siRNA complexed with myr-R9-LyP-1 and E9 peptides. MDA-MB-231 cells were either left untreated or incubated with control siRNA or a combination of Stat3 and c-Myc siRNAs complexed with either Lf-2000 or myr-R9-LyP-1 and E9 peptides. After 24 hours, the cells were replated in soft agar. After 2 weeks, cell colonies larger than 50 cells were quantitated and the individual results from triplicate plates are shown. The results shown in Fig 9 are derived from these experiments.
(TIF)
