# Supplementary figures and images for: Delivery of gene targeting siRNAs to breast cancer cells using a multifunctional peptide complex that promotes both targeted delivery and endosomal release
Source: PLoS One. 2017 Jun 30;12(6):e0180578. doi: 10.1371/journal.pone.0180578 (PMC5493434; doi:10.1371/journal.pone.0180578)

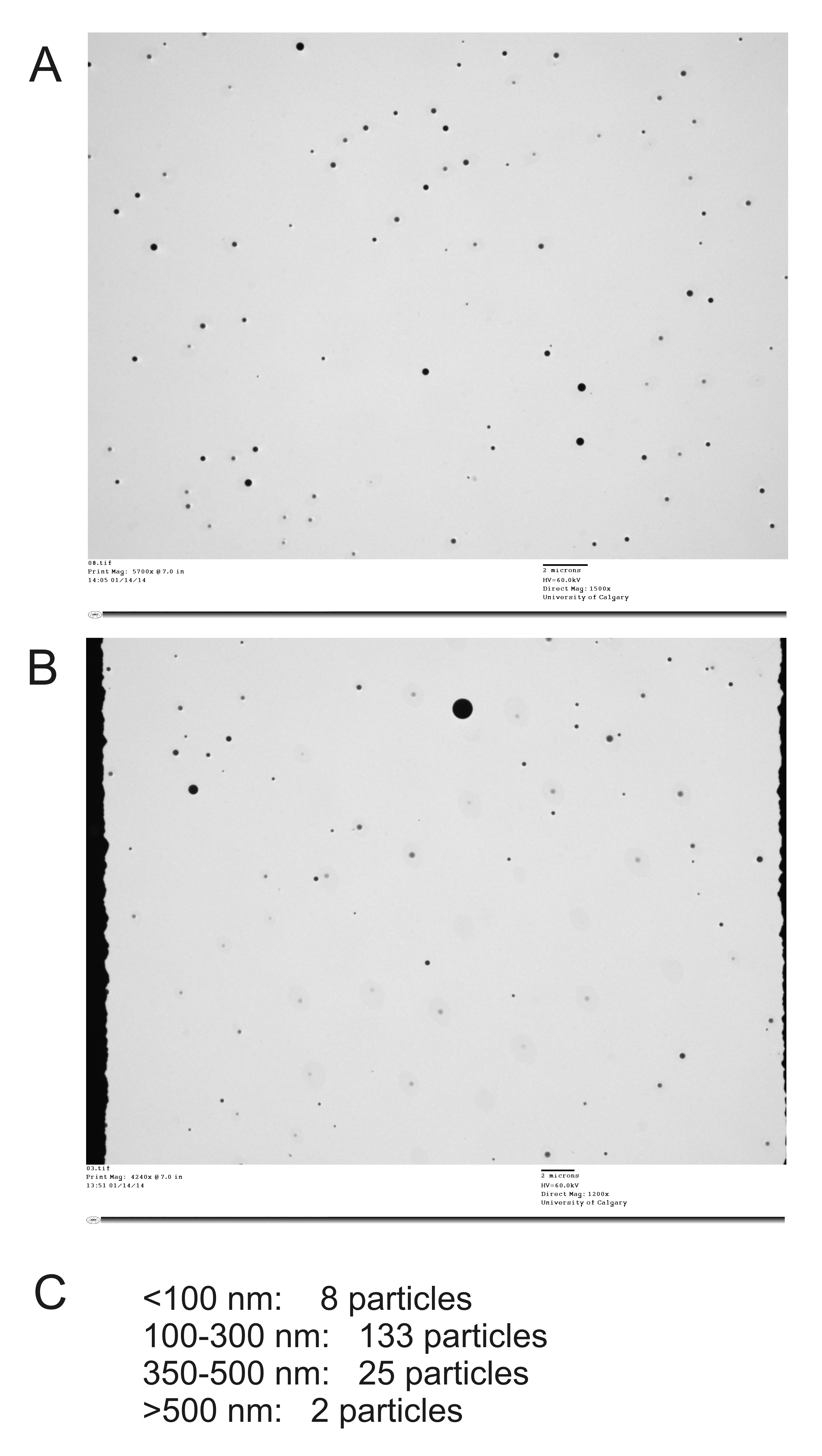

Supplement: S1 Fig — The peptide/siRNA complexes were stained with uranyl acetate and were visualized on a transmission electron microscope. Two fields at A) 1500X magnification and B) 1200X magnification were photographed and these pictures are shown. The particles from the 2 images were measured and the numbers of particles that fell within specified size ranges reported (C). The particle size distribution shown in Fig 4 is derived from the measurement of the particles from these two photographs. (TIF) [file pone.0180578.s001.tif]

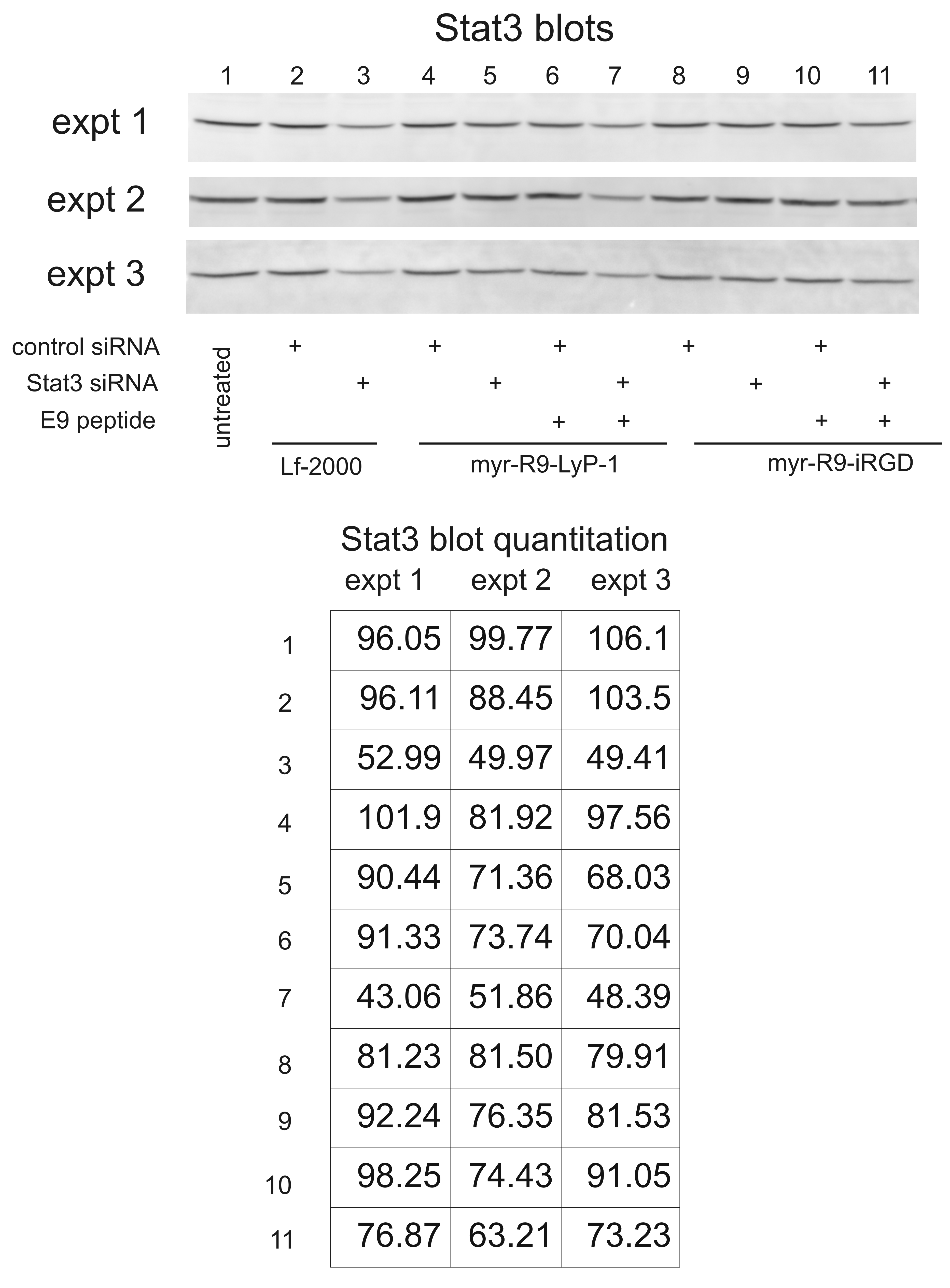

Supplement: S2 Fig — Peptide/siRNA complexes were incubated on the cells overnight. After 48 hours, cell extracts from the MDA-MB-231 cells were examined for Stat3 protein expression by Western blotting. The resulting blots from triplicate experiments are shown (upper panel) and the results from quantitation of the blots (lower panel). The results shown in Fig 7 are derived from these 3 experiments. (TIF) [file pone.0180578.s002.TIF]

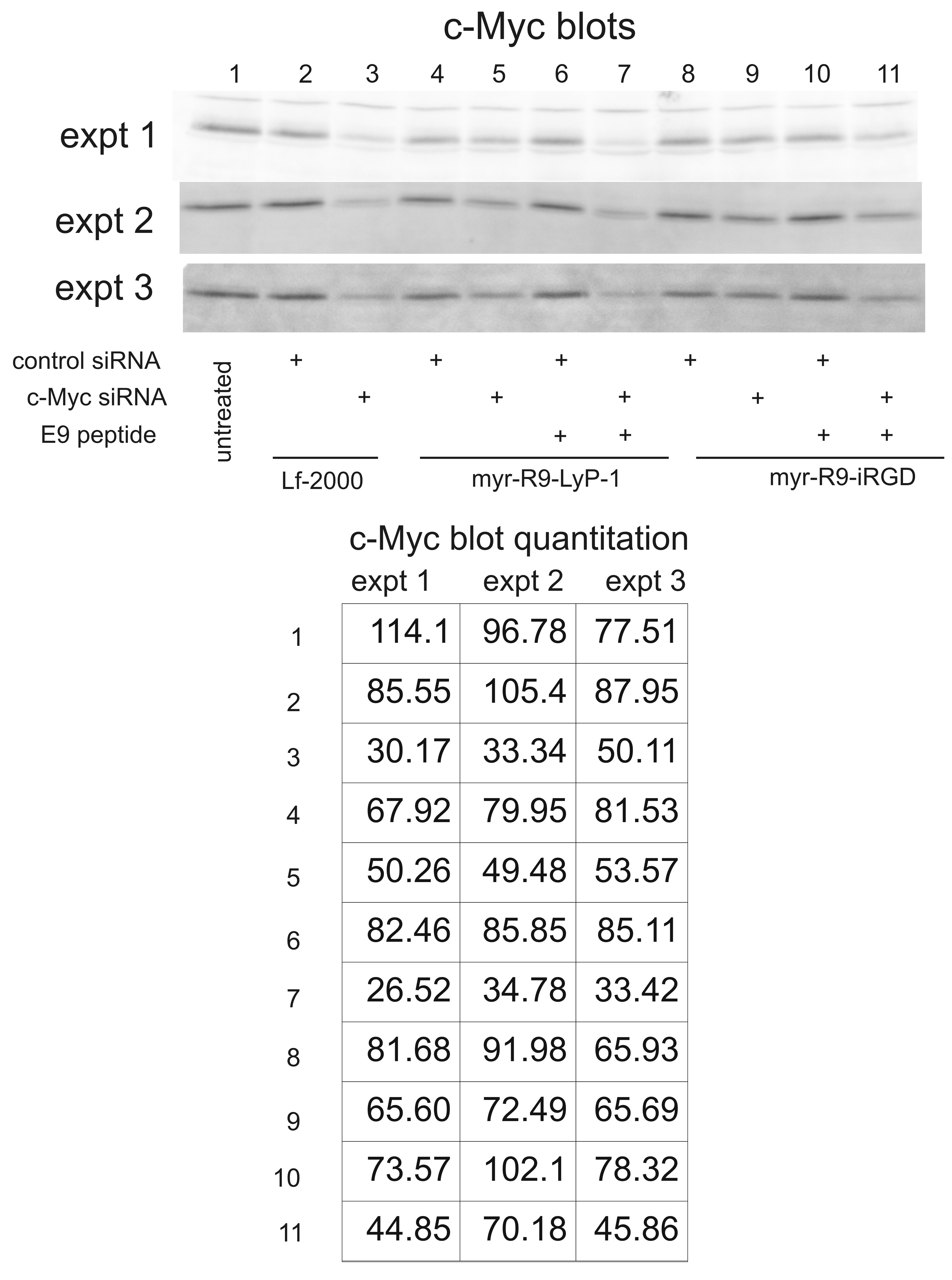

Supplement: S3 Fig — Peptide/siRNA complexes incubated on the cells overnight. After 48 hours, cell extracts from the MDA-MB-231 cells were examined for c-Myc protein expression by Western blotting. The resulting blots from triplicate experiments are shown (upper panel) and the results from quantitation of the blots (lower panel). The results shown in Fig 8 are derived from these 3 experiments. (TIF) [file pone.0180578.s003.tif]

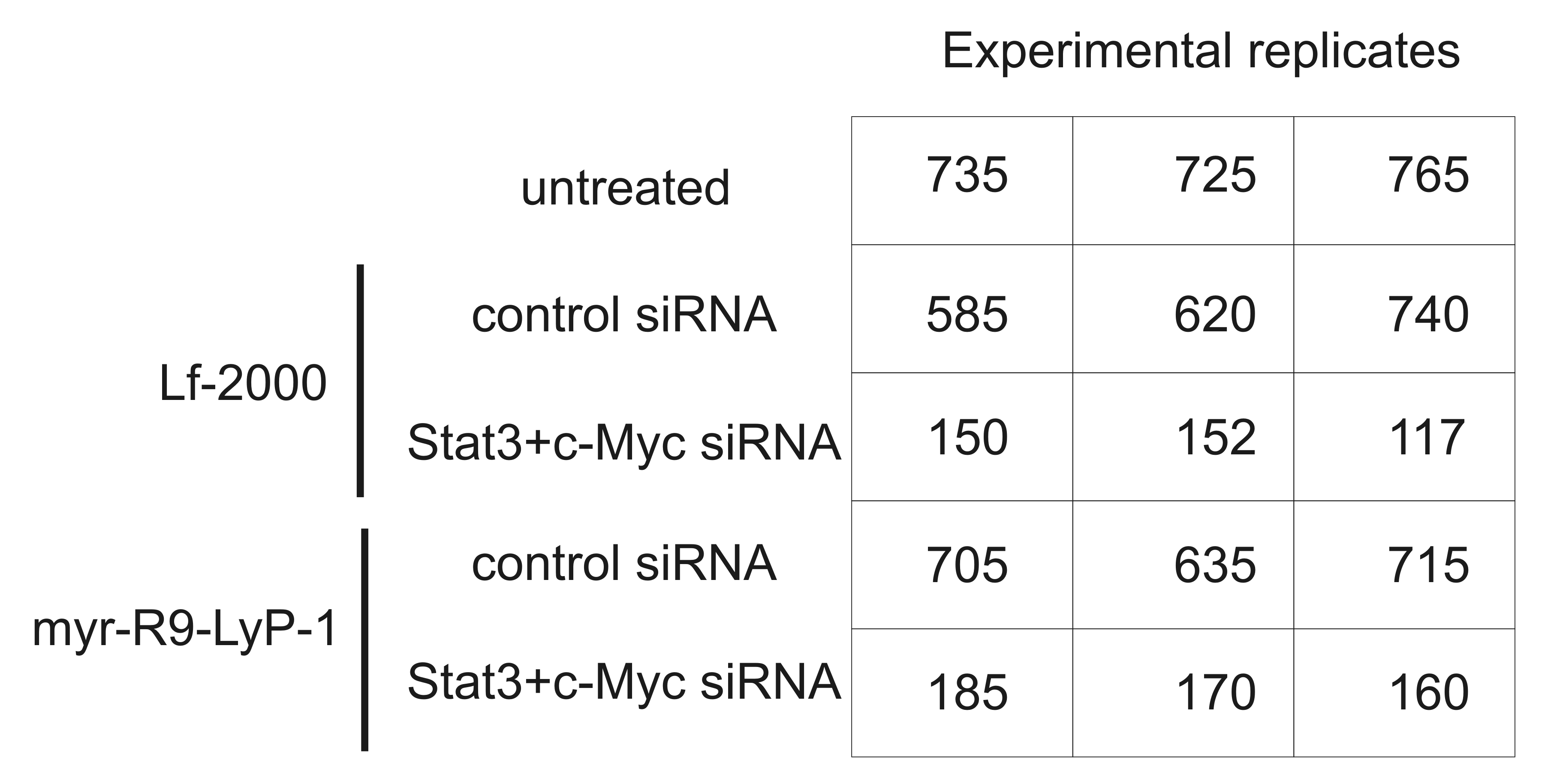

Supplement: S4 Fig — MDA-MB-231 cells were either left untreated or incubated with control siRNA or a combination of Stat3 and c-Myc siRNAs complexed with either Lf-2000 or myr-R9-LyP-1 and E9 peptides. After 24 hours, the cells were replated in soft agar. After 2 weeks, cell colonies larger than 50 cells were quantitated and the individual results from triplicate plates are shown. The results shown in Fig 9 are derived from these experiments. (TIF) [file pone.0180578.s004.tif]
